# The advancement and utility of multimodal imaging in the diagnosis of degenerative disc disease

**DOI:** 10.3389/fradi.2025.1298054

**Published:** 2025-03-06

**Authors:** Eric M. Teichner, Robert C. Subtirelu, Connor R. Crutchfield, Chitra Parikh, Arjun Ashok, Sahithi Talasila, Victoria Anderson, Milan Patel, Sricharvi Mannam, Andrew Lee, Thomas Werner, William Y. Raynor, Abass Alavi, Mona-Elisabeth Revheim

**Affiliations:** ^1^ Sidney Kimmel Medical College, Thomas Jefferson University, Philadelphia, PA, United States; ^2^ Department of Radiology, Hospital of the University of Pennsylvania, Philadelphia, PA, United States; ^3^ Department of Radiology, Rutgers Robert Wood Johnson Medical School, New Brunswick, NJ, United States; ^4^ The Intervention Center, Division of Technology and Innovation, Oslo University Hospital, Oslo, Norway; ^5^ Faculty of Medicine, Institute of Clinical Medicine, University of Oslo, Oslo, Norway

**Keywords:** degenerative disc disease (DDD), magnetic resonance imaging (MRI), positron emission tomography/computed tomography (PET/CT), early diagnosis, artificial intelligence (AI), imaging technologies, pathophysiology, treatment strategies

## Abstract

Degenerative disc disease (DDD) is a common spinal condition characterized by the deterioration of intervertebral discs, leading to chronic back pain and reduced mobility. While magnetic resonance imaging (MRI) has long been the standard for late-stage DDD diagnosis, its limitations in early-stage detection prompt the exploration of advanced imaging methods. Positron emission tomography/computed tomography (PET/CT) using ^18^F- fluorodeoxyglucose (FDG) and ^18^F-sodium fluoride (NaF) has shown promise in identifying metabolic imbalances and age-related spinal degeneration, thereby complementing CT grading of the disease. The novel hybrid imaging modality PET/MRI provides new opportunities and are briefly discussed. The complex pathophysiology of DDD is dissected to highlight the role of genetic predisposition and lifestyle factors such as smoking and obesity. These etiological factors significantly impact the lumbosacral region, manifesting in chronic low back pain (LBP) and potential nerve compression. Traditional grading systems, like the Pfirrmann classification for MRI, are evaluated for their limitations in capturing the full spectrum of DDD. The potential to identify early disease processes and predict patient outcomes by the use of artificial intelligence (AI) is also briefly mentioned. Overall, the manuscript aims to spotlight advancements in imaging technologies for DDD, emphasizing their implications in refining both diagnosis and treatment strategies. The role of ongoing and future research is emphasized to validate these emerging techniques and overcome current limitations for more effective early detection and treatment.

## 1 Introduction

**Figure 1:**
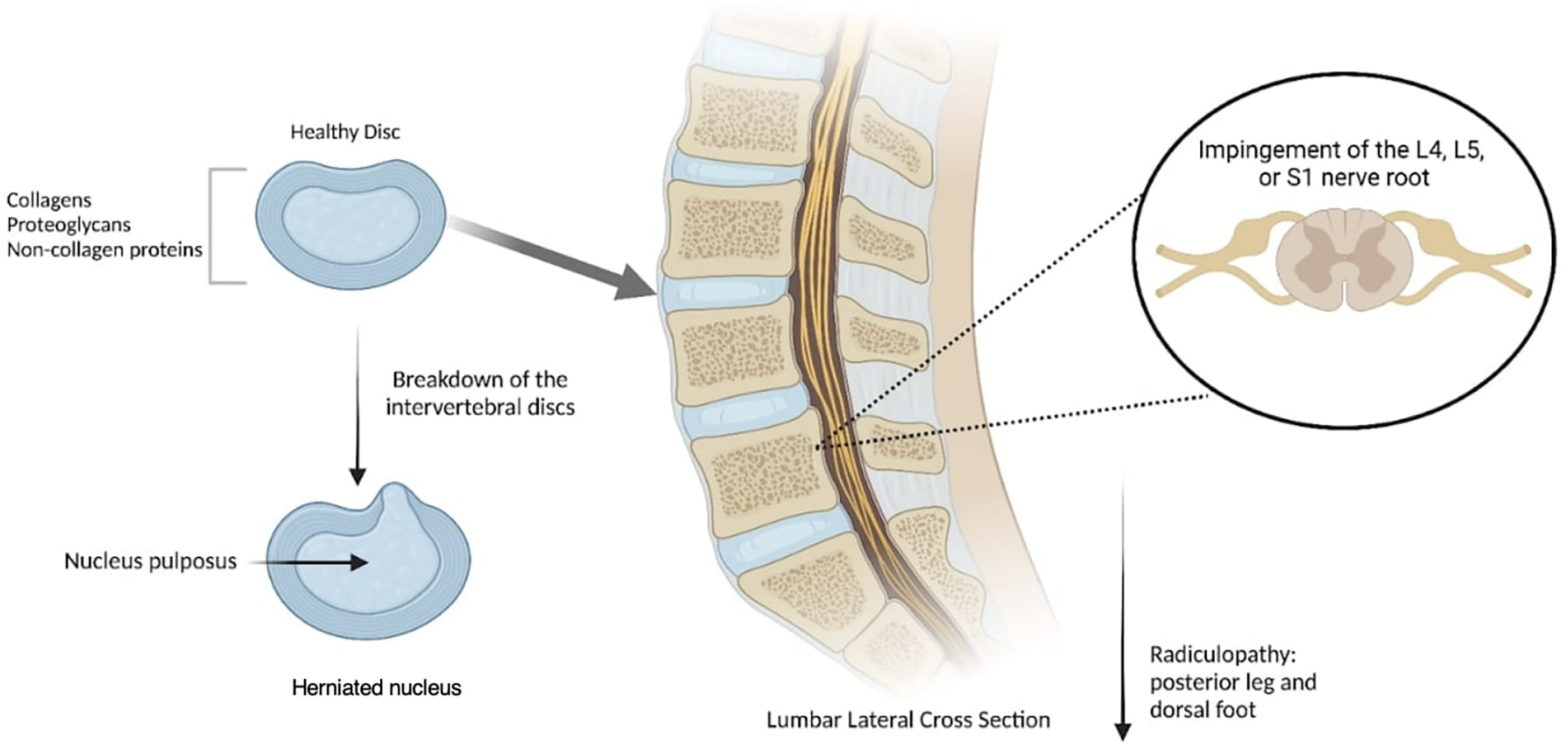
Degenerative disc disease (DDD) occurs when spinal discs are herniated and the nucleus pulposus is protruded due to mechanical stress, resulting in a compromised structure that induces pain radiating downward. Created with Biorender.

Intervertebral discs serve as cushions of fibrocartilage located between each vertebral body of the spine. Their primary functions are to provide structural support to the spine and to act as shock absorbers, thereby preventing the vertebral bodies from grinding against each other (1). These discs consist of two layers: an inner soft structure known as the nucleus pulposus, and an outer firm layer called the annulus fibrosus (1). Normal intervertebral disc structure can be disrupted, initiating a degenerative cascade marked by an imbalance between catabolic and anabolic processes within the discs (2). This imbalance leads to extracellular matrix degradation, neo-innervation, and neovascularization, culminating in disc degeneration. Known as degenerative disc disease (DDD), this degeneration arises from various factors such as mechanical stress, trauma, genetics, or nutritional imbalances (3). The progression of DDD can result in disc herniation of the nucleus pulposus, often manifesting as chronic pain (1, 3) (Figure 1). In fact, DDD is one of the primary causes of chronic lower back pain (3), with over 90% of herniated discs occurring in the lumbosacral region. These herniations commonly take place at the L4-L5 or L5-S1 disc spaces, leading to impingement of the L4, L5, or S1 nerve roots (1). Such impingements result in radiculopathy extending into the posterior leg and dorsal foot (1).

Imaging techniques have proven invaluable in detecting disc pathology, identifying degenerative disc changes, and observing the consequences of spinal instability. Different imaging modalities can be performed, such as magnetic resonance imaging (MRI), ultrasonography, computed tomography (CT), plain radiographs, and positron emission tomography (PET). Among these, magnetic resonance imaging is currently the most commonly used and accepted modality for diagnosing DDD (4). PET using ^18^F-fluorodeoxyglucose (FDG) conveys glucose metabolism and is highly sensitive for the early detection of inflammatory processes (5), and PET using 18F-sodium fluoride (NaF) demonstrates bone turnover such as during reactive bone formation (6), which are features present in DDD.

MRI and PET are both molecular imaging modalities and may therefore have the potential to provide evidence for pathological molecular changes before structural changes. In this review, “MRI” refers to conventional techniques unless specified otherwise (e.g., quantitative MRI or advanced techniques). We aim to further emphasize the importance of multimodal imaging in the diagnosis of DDD.

## 2 Understanding degenerative disc disease (DDD)

DDD is a common condition characterized by the deterioration of intervertebral discs in the spine. Although often considered a natural part of the aging process, specific factors such as genetics, trauma, and lifestyle choices can accelerate its progression (7). The pathology of DDD involves the gradual breakdown of the intervertebral discs, which serve as cushions between the vertebrae, providing flexibility and shock absorption. As these discs age and experience wear, they lose water content and structural integrity, leading to decreased disc height and compromised function. This degeneration can also result in the formation of disc bulges or herniations, causing compression of adjacent nerves and spinal instability (8).

The progression of DDD is generally slow and can vary among individuals. Initial symptoms often include minor back pain or stiffness, which may worsen over time. As the condition progresses, individuals may experience chronic pain, symptoms radiating into the arms or legs, muscle weakness, and reduced mobility (9). Numerous studies have explored the underlying mechanisms of DDD, such as genetic predisposition, mechanical stress, inflammation, and biochemical changes within the disc. Research has identified various biomarkers and molecular pathways associated with disc degeneration, offering potential targets for future therapeutic interventions (10).

DDD particularly impacts the lumbosacral region, encompassing the lower back and sacrum. Due to its biomechanical characteristics and the stresses placed upon it, this area of the spine is especially vulnerable to disc degeneration (10). In the lumbosacral region, degenerative changes occur over time in the intervertebral discs between the lumbar vertebrae and the sacrum. As these discs lose hydration and structural integrity, their ability to absorb shock and provide stability diminishes, leading to a range of symptoms and functional limitations (11). Individuals with DDD in this region commonly experience lower back pain, which may either be localized or radiate down the buttocks and legs. Pain often becomes chronic and worsens with movement, prolonged sitting or standing, or activities involving bending or lifting. If degenerated discs result in spinal canal stenosis or nerve root impingement, additional symptoms like leg pain, numbness, or weakness may occur (12). The impact of DDD on the lumbosacral region can significantly affect an individual's quality of life, limiting their capacity for daily activities and work-related tasks (13).

Several factors can contribute to the progression of DDD. Genetics plays a significant role; certain gene variants have been linked to an increased susceptibility to disc degeneration (14). Lifestyle factors like smoking, obesity, and sedentary behavior can also accelerate the progression of DDD. Smoking, for example, has been shown to impair disc health by reducing blood supply and nutrient delivery to the discs. Similarly, obesity and sedentary behavior contribute to increased mechanical stress on the spine, accelerating disc degeneration (15). These findings highlight the importance of genetic predisposition and lifestyle modifications in managing and potentially slowing the progression of DDD.

It has been noted that up to 84% of people have back pain at some point in their lives (16) with low back pain (LBP) specifically being one of the world's leading causes of disability, affecting nearly 12 million people in the U.S (17) and disproportionately affecting aging and elderly populations (24, 25). Genetic predisposition also has important contributions to the pathophysiology of DDD and may make its development virtually inevitable in some individuals (3, 18–21).

In terms of epidemiology, one cross-sectional MRI study of 1,043 volunteers between the ages of 18–55 found that degenerative disc changes were present in 40% of individuals under 30, with the prevalence of lumbar changes increasing progressively to over 90% by 50–55 years of age. The authors also found a positive correlation between DDD severity and low back pain (22). It is worth noting that rates of DDD may also be generally underreported due to the known high prevalence of asymptomatic intervertebral disc (IVD) disease (17, 23, 24). With an aging population and the prevalence of sedentary lifestyles in the U.S., the health concerns of DDD and its sequelae will likely continue to rise, along with direct and indirect costs previously reported to exceed $50 billion in healthcare expenditures (17) and $100 billion in related resources annually (20).

## 3 Pathophysiology of degenerative disc disease

Intervertebral discs are composed mainly of collagens (50%–70%), water-binding proteoglycans (10%–50%), and other non-collagenous proteins (<25%) that provide the disc with height, force distribution properties, and tensile strength (25). The pathophysiology of DDD is poorly understood; however, its progression is marked by increased hypoxia, inflammation, neoinnervation and neovascularization, accelerated catabolism, collagen breakdown, and reduced glycosaminoglycan and water content in the extracellular matrix (ECM) (3, 18, 20). The initial stages of the disease are characterized by a metabolic imbalance between catabolic and anabolic processes in the IVD that are accompanied by an ingrowth of nerves, vasculature, and granulation tissue (9). Ongoing inflammation also introduces a deleterious feedback loop, wherein responding immune cells produce cytokines and chemokines that not only recruit more cells into the disc but also upregulate the expression of matrix degradative enzymes (3). These changes then give way to a decreased production of ECM components, mainly proteoglycans and their hydrophilic glycosaminoglycan branches in the nucleus pulposus. Such losses of the hypertonic milieu reduce the disc's capacity to bind water and reduce fluid pressurization, thereby also impairing mechanical function and load resistance (17–19). Together, these processes generate abnormal collagen orientation and deposition of a calcified layer within the cartilaginous endplate in the more advanced stages of DDD (19, 26). As a result, diseased discs experience lamellar disruption, eventually leading to loss of tensile strength and compromised compressive force transmission to the annulus fibrosus (17, 18, 26). Degenerative changes at the cellular level ultimately yield gross changes in morphology such as disc height reduction and disc-space narrowing, disc bulging, endplate irregularities, annular rim tears, and osteophyte formation (17–19). Degenerative changes in the discs are often associated with structural disruptions such as annular fissures or herniations. Annular fissures describe separations in the annular fibers without implying a traumatic origin. Herniations, on the other hand, represent a focal displacement of disc material beyond the disc space, classified into protrusions, extrusions, or sequestrations depending on the extent of displacement and continuity with the parent disc (27). Paraspinal muscle atrophy, particularly involving the multifidus muscle, has emerged as an important factor in the progression of DDD. Studies have shown that atrophy of the multifidus correlates with accelerated degeneration of intervertebral discs, as well as degeneration of endplates and facet joints. This atrophy can result from chronic disuse, age-related changes, or direct neural compromise, leading to a reduction in spinal stability and increased mechanical stress on the discs and adjacent structures (28). The interplay between muscle health and spinal degeneration underscores the importance of including paraspinal muscle evaluation in the clinical assessment of DDD, particularly in symptomatic patients. Future research into therapeutic interventions, such as physical therapy targeting paraspinal muscle strength, may prove valuable in slowing disease progression and improving patient outcomes. These distinctions are critical for correlating imaging findings with potential clinical symptoms and guiding treatment strategies. It is important to understand the pathophysiology of DDD to understand the utility and shortcomings of the current diagnostic imaging modalities.

## 4 Traditional imaging techniques for degenerative disc disease (DDD)

In a workup of LBP, radiographs are the first investigative measure and anterior-posterior and lateral views are routine and predominantly used for the evaluation of frontal and sagittal balance, respectively (30, 31). On x-ray (XR), the advanced stages of DDD are more easily seen as they involve gross bony changes, and typical signs including disc height reduction, joint space narrowing, osteophyte formation, facet hypertrophy/arthrosis, and alterations in articular alignment can be readily identified (18, 29). Dynamic XR testing can also be used to evaluate for the presence of hypermobility and instability, defined as a 4 mm segmental dislocation or >10–12 degrees of the angular fold, and spot film XR can help further characterize of the degree of degenerative changes present at specific location (31). Discs are categorized based on pathology, such as normal, degeneration, or herniation. Herniated discs are further classified as protrusion or extrusion, with protrusions defined by a broader base of displaced material relative to its height, while extrusions show a more pronounced displacement with a narrower connection to the disc space of origin. Such standardized nomenclature ensures clarity in imaging-based diagnosis and clinical communication (27). While XR provides an abundance of useful morphological information about the spine and IVDs, mainly through spinal curvature and alignment and vertebral outlines, it cannot fully appreciate the complex underlying pathological processes of DDD mentioned in the discussion above, including water content, metabolic derangement, proteoglycan composition, and inflammatory processes. Standing XR, which spine surgeons use to plan surgery, is considered the gold standard for measuring spinal alignment and there is ongoing debate about imaging the spine in a supine position to negate the influence of gravity for disc height, but nevertheless, the limitations remain the same. Most importantly, perhaps, radiography in general carries with it exposure to ionizing radiation, which is well known to increase the risk of health problems such as altered immunity and an increased risk of cancer (32).

Similar to plain films, computed tomography (CT) can help assess the osseous changes of the spine with the expanded capacity to evaluate the anterior-posterior dimension of the spinal canal, hypertrophy of the ligamentum flava, intervertebral joint spaces, as well as protrusions of the intervertebral discs (30). Modern three-dimensional (3D) reconstructions further extend the benefits of CT by producing a multi-axis visual of the spine, which allows for a more exact diagnosis of spinal stenosis, dislocation of the nucleus pulposus, bony fracture, and certain ligamentous ossification (30). In addition, computed tomography-osteoabsorptiometry (CT-OAM) is a technique based on conventional CT scans that displays the mineral density distribution of the subchondral bony endplate as a surface-color map (26). As previously discussed, the more advanced stages of DDD cause deposition of a calcified layer within the endplate that disrupts the attenuation of compressive forces through the IVD. By producing a color gradient which corresponds with increasing mineral density, CT-OAM can more effectively describe DDD progression, wherein the vertebrae of degenerated spines show a broader outer ring of endplate mineralization that extends inward (26). While used widely, CT technology does have notable limitations. Firstly, like XR, CT exposes patients to radiation, but at a larger dose. Patient exposures should therefore be chosen judiciously. It is also commonly affected by imaging artifacts, particularly at the cervicothoracic junction, and by the inability to differentiate the content of the spinal canal and the outline of the dural sac without the help of intrathecal contrast administration (33). For these reasons, CT is often limited to situations of diagnostic uncertainty or surgical planning.

Myelography can also be a useful technique as an adjunct to CT, especially if constrictions of the spinal canal that would block the flow of the contrast medium are suspected (17, 30). For this reason, the use of contrast provides more specificity for disc herniation when compared to CT, as obliteration of the epidural space is more easily appreciated on an axial view with interruption of the contrast's hyperdense signal (33). Inflammatory and neoplastic tissues are also more easily visualized with contrast medium and can be used to distinguish a recurrent herniation by the presence of increased vascularization (33). Importantly, myelography remains relevant to the postoperative examination as well since the contrast-filled thecal sac presents arachnoid adhesions, CSF leaks, and epidural hemorrhage more clearly without being obscured by metal-induced artifacts to the degree that CT is (33). Myelography, while historically a cornerstone imaging technique for spinal disorders, has significantly declined in use with the advent of magnetic resonance imaging (MRI). MRI's superior soft tissue resolution and non-invasive nature have largely replaced myelography in the diagnostic flow-chart for DDD. The limitations of myelography include its invasive nature, risks of meningeal irritation, post-myelography headaches, and limited ability to evaluate soft tissue structures comprehensively (33). These factors, combined with the widespread availability and diagnostic utility of MRI, have relegated myelography to a supplementary role in cases where MRI is contraindicated or insufficient, such as in evaluating certain postoperative conditions or in the presence of metal artifacts.

Finally, though the use of nonionic contrast media has limited meningeal irritation and neurotoxicity and reduced the morbidity of myelography, the risks of iatrogenic injury and post-myelography meningitis remain (33), and so it is performed in very select cases and not widely used for imaging DDD (30). It is also worth recognizing that the volume and speed of absorption of contrast agents into the IVD are limited by the disc's inherent transport processes (19) an all contrast-enhanced imaging limited should be interpreted with these temporal restrictions in mind.

In the diagnosis of DDD, MRI is the modality of choice when conservative management fails and is considered the best non-invasive method to study IVD degeneration (3, 18, 34, 35). Preferred for its excellent soft-tissue resolution and lack of ionizing radiation (18, 33, 35), MRI has a superior ability to differentiate sub-disc regions, endplates, facet joints, ligaments, and nerves when compared to radiography and CT (3, 18, 34, 35). It can also provide information about disc dehydration, proteoglycan loss, and collagen breakdown, all of which are key in describing the progression of DDD (3). Building on this foundation, emerging advanced MRI techniques like T1ρ imaging, T2 relaxation mapping, and sodium MRI offer a novel approach by detecting early biochemical changes, such as proteoglycan depletion, before structural damage becomes evident. These advancements not only complement conventional MRI but also pave the way for earlier diagnosis and targeted treatment strategies, marking a significant progression in imaging for DDD.

MRI plays a critical role in detecting and grading spinal central and lateral stenosis, conditions often associated with DDD. Central canal stenosis, caused by factors such as disc herniation, ligamentum flavum hypertrophy, or osteophyte formation, is commonly assessed using the Schizas classification (36). This system grades stenosis based on the morphology of the dural sac and the extent of nerve root compression, providing a standardized framework for evaluating severity. Similarly, lateral stenosis, which involves narrowing of the neural foramina and impingement on exiting nerve roots, is often classified using the Lee system for foraminal dimensions and nerve compression (37). An extended Lee classification further delineates central canal narrowing (38), while the Spinnato classification provides a comprehensive approach that integrates central and lateral stenosis with associated pathologies and symptomatology (39). These classification systems are instrumental in guiding treatment decisions, including determining the need for surgical intervention, and highlight the value of MRI in the precise evaluation of DDD-related spinal stenosis. As a result, MRI also offers better categorization of degenerative and non-degenerative diseases than other imaging modalities, which can be helpful when the differential is broad (33). The standard MRI protocol for DDD typically includes sagittal T1-weighted (T1w) fast spin-echo (FSE), sagittal T2-weighted (T2w) FSE, and axial T2w FSE images (18, 24). When imaging the whole spine, most institutions will also add a short tau inversion recovery (STIR) sequence, a gradient echo sequence (particularly in the cervical spine) and a coronal proton density-weighted (PDw) sequence (24). Notably, T2w sequences are effective in imaging canal and foraminal stenosis, IVD dehydration, collagen sequence breakdown, and loss of proteoglycans (and moisture content), while STIR is sensitive for early fractures and inflammation (3, 40). Additional sequences can be added on as needed such as axial T1w sequences, sagittal fat-suppressed T2w sequences, and gadolinium-enhanced T1w sequences (especially for tumors, infections, and the postoperative spine) (18).

**Figure 2:**
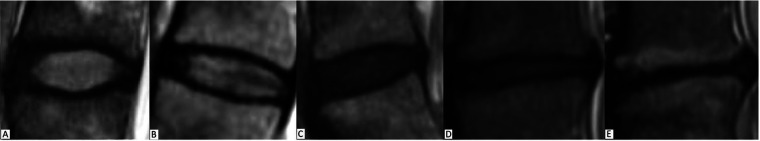
Pfirrmann grading system for disc degeneration on sagittal T2 weighted images. **(A)** Grade I, bright and homogeneous disc with clear distinction between nucleus pulposus and annulus fibrosis. Normal disc height. **(B)** Grade II, inhomogeneous disc with horizontal dark band. Nucleus and annulus are clearly differentiated. Preserved disc height. **(C)** Grade III, dark disc with unclear distinction between nucleus and annulus. Disc height is usually normal. **(D)** Grade IV, dark and heterogeneous disc with decreased disc height. **(E)** Grade V, dark and collapsed disc with no distinction between the nucleus and annulus. Reproduced with permission from Abdalkader et al., (77).

For interpreting the MRI in the setting of DDD, the Pfirrmann classification is the most accepted grading scale and is used exclusively in T2w images (18). The classification grades DDD on a scale of I (best) to V (worst) based on disc structure, disc height, quality of distinction between the nucleus pulposus and annulus fibrosus, and T2 signal intensity, where a reduction in signal of the nucleus pulposus, increased heterogeneity, and loss of disc height indicate more advanced disease (grades IV-V) (Figure 2) (18, 41). Another common method of classification was first proposed by Modic et al. in 1988 (42), and focuses on fibrovascular replacement of the hematopoietic marrow and IVD endplate signal changes secondary to edema and inflammation (23). The Modic classification can be used in T1w and T2w images, with higher scores representing greater changes (42). It is important to note that the Modic classification evaluates dynamic pathological processes, some of which have been shown to be reversible (43), and should be interpreted accordingly when describing a disease state. Although the Modic and Pfirrmann classifications are the most commonly used MRI scoring systems for DDD, some evidence suggests that MRI findings consistent with high-grade scores do not necessarily correlate with intensity or progression of chronic LBP, further obscuring the already poorly understood relationship between our current means of interpreting imaging and the clinical symptoms of DDD (3, 24).

Furthermore, MRI is less sensitive to the early changes of DDD occurring at the cellular level^2^ and most of the changes observed on imaging are a consequence of aberrant physiology that has been developing for months to years prior (19). This is likely because the IVD has a very low cell count with a slow turnover of the components in the ECM (19). Despite the dynamic solute exchange and metabolic processes that occurs at the cellular level of the disc when under stress (18), conventional MRI techniques offer limited insight into the ongoing processes that underly DDD progression. In the same vein, authors have suggested that the current classification of “non-specific chronic low back pain,” an umbrella term under which patients with pain that does not correlate with observed anatomical distortion are described, is a vague and insufficient diagnosis that would benefit from more advanced imaging that can better describe the disease processes underlying the source of pain (44). Patients with chronic LBP are a heterogeneous group with varying pathology that often includes DDD, but current imaging modalities are insufficient in identifying all of the causal, particularly metabolic, mechanisms of the pain. It is for these reasons, that investigators are studying new imaging techniques that involve more sensitive analysis of live cell and tissue behavior not visualized with current techniques. In doing so, future imaging may bolster preventative measures of DDD by providing a deeper understanding of its causes and allowing earlier diagnosis, rather than intervening at the later stages of disease. Developing quantitative MRI (QMRI) techniques such as T2 star (T2*) mapping, T1ρ and T2 relaxation mapping with and without magic echo sequences, sodium MRI, delayed gadolinium-enhanced MRI of cartilage (dGEMRIC), glycosaminoglycan chemical exchange saturation transfer (gagCEST), MR Spectroscopy (MRS), and PET/CT are emerging technologies able to evaluate disc quality based on biochemical composition, proteoglycan content, and metabolism that have already begun to help address current shortcomings. Emerging imaging techniques have demonstrated significant improvements over traditional methods, particularly in early detection of DDD. For example, quantitative MRI techniques like T2 relaxation mapping and T1ρ imaging offer enhanced sensitivity for identifying biochemical changes, such as proteoglycan loss and dehydration, before structural damage becomes visible on conventional MRI. T2* mapping enables better noise reduction and shorter acquisition times, making it a valuable tool for clinical workflows. Similarly, sodium MRI directly measures ^23^Na^+^ ion concentrations as a correlate for proteoglycan content, providing unique insights into the early stages of disc degeneration. PET/CT, using tracers such as FDG and NaF, offers the ability to detect metabolic activity and bone turnover that precede gross anatomical changes visible on MRI or CT. These advancements collectively allow for earlier and more precise diagnoses, enabling timely interventions that could slow disease progression.

## 5 Advanced imaging techniques for degenerative disc disease (DDD)

### 5.1 Quantitative magnetic resonance imaging (QMRI) techniques

#### 5.1.1 T2 star (T2*) mapping

The main advantages of T2* mapping over conventional T2 imaging are its three-dimensionality, shorter acquisition time, and better noise reduction (29, 45). This technique uses T2* relaxation times to image the architecture of the macromolecule matrix and water movement of cartilage, crucial components of IVD physiology. Quantitative T2* relaxation time mapping is sensitive in assessing features of disc degeneration and has been shown to predict altered functional states of the lumbar spine better than traditional Pfirrmann grading, where a faster relaxation time correlates with a higher degree of cartilage degeneration (45). Like many of these novel QMRI studies, however, T2* mapping does require high magnetic strengths and radiofrequency pulse energy levels and much of the research validating T2* imaging of the spine uses a 3 Tesla magnet.

#### 5.1.2 T1ρ and T2 relaxation mapping

T1ρ and T2 mapping techniques digitize water molecule dispersion within the cartilaginous matrix, which generates observable tissue contrast based on the unique variations in IVD protein content, specifically, the glycosaminoglycan content of proteoglycans (17, 19, 34, 46). Because disc dehydration secondary to proteoglycan loss is a key component of DDD, both techniques offer more sensitive evaluation of IVD composition and the degree of cartilage degeneration compared to traditional MRI, with T1ρ-weighted being more sensitive than T2 mapping (17, 46). T2 and T1ρ-weighted imaging can also exploit the unique relaxation time constants of the various tissues in the spine, such as the annulus fibrosus and nucleus pulposus, through different imaging parameters – repetition time (TR), echo time (TE), and spin-lock time (TSL) – to achieve good tissue resolution (17). T1ρ imaging in particular carries superior advantages by generating more contrast between cartilage, fluid, and other joint structures with a high level of accuracy and precision (17). Also important to both T2 and T1ρ is their accessibility, as (1) neither technique requires special preparation, contrast agent administration, or specific hardware and (2) the pulse sequences and software for generating their quantitative maps are now available in commercial packages (15). It is worth noting, however, that T1ρ requires an additional RF pulse sequence, leading to a higher specific absorption rate (18). Finally, a still emerging T2 pulse sequence termed “magic echo” has shown promise in boosting the sensitivity of T2 relaxation mapping for degenerative disc changes by elongating relaxation times compared to conventional spin echo. Early evidence suggests that this sequence provides T2 mapping studies greater dynamic range for biochemical changes and may eliminate imaging artifacts caused by dipole-dipole interactions between water molecules (17). With further development, this technique may be useful for acquiring images of tissues with short T2 relaxation times, thereby further enhancing the sensitivity of existing T2 relaxation mapping. Applied to the evaluation of DDD, the outlined elements of T1ρ and T2 mapping make them highly attractive due to their potential for routine use in the clinical setting and ability to detect early degenerative changes before any gross morphological change manifest (19, 29, 46).

#### 5.1.3 Sodium MRI

Sodium MRI relies on imaging the nuclei of ^23^Na^+^ ion content in tissues, rather than the proton nuclei of water. Compared to most tissues, IVDs have increased levels of ^23^Na^+^ ions in the ECM, making this study particularly relevant to the evaluation of DDD. Indeed, *in vivo* studies have demonstrated the use of sodium MRI has a highly accurate and specific modality for evaluating the sodium content of IVDs, articular cartilage, cardiac muscle, and brain tissue (46–51). Sodium MRI has also been shown to be a validated tool in quantifying water content, using sodium concentrations as a correlate to the amount of proteoglycans present. In a 2010 study of sodium MRI, the authors used specimens of bovine cartilage to assess the correlation of sodium with proteoglycan content and found a significant linear regression (*r* = 0.71, *p* < 0.05) between the two, with the highest sodium content in the nucleus pulposus (52). Using a 3 T scanner, sodium agarose phantom, and a T2w MRI for comparison, they also successfully implemented the technique *in vivo* with a small-scale feasibility study of two young male subjects. Here, they found that the discs of the symptomatic subject showed a significantly decreased ^23^Na^+^ concentration compared to those of the asymptomatic subject (48).

However, sodium MRI is not without limitation. One of which that is well known is its long scanning times. Because there is a lower natural abundance of ^23^Na^+^ in the human body compared to proton concentrations, scans employ fast pulse sequences that must be averaged over time (52). Another is that the images of sodium MRI lack the spatial resolution of other MRI techniques, however, continuing research and the increasing prevalence of more powerful 3 T and 7 T magnets could refine the signal-to-noise-ratio of images and transform the power of this technique to increase its potential for mainstream clinical use. Intra- and extracellular sodium concentrations are increasingly becoming reliable biomarker of proteoglycan content and as such have major potential for the early diagnosis of DDD. While it will likely remain an adjunct modality to conventional proton MRI, sodium MRI offers valuable information about the metabolic processes behind disc degeneration (53).

#### 5.1.4 Glycosaminoglycan chemical exchange saturation transfer (GagCEST)

**Figure 3:**
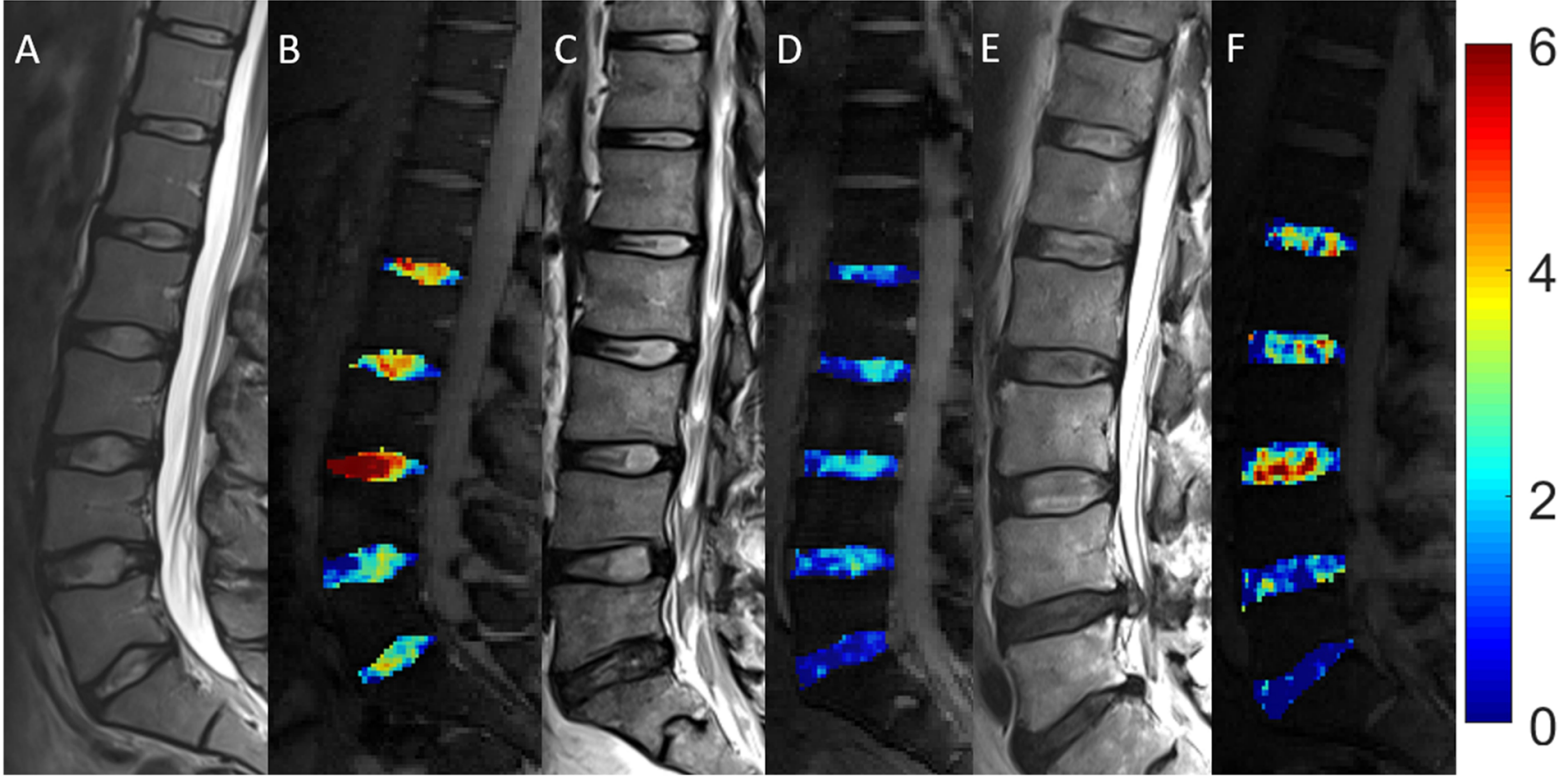
Morphologic and compositional imaging findings of lumbar intervertebral discs of an asymptomatic volunteer **(A,B)**, a patient with nonspecific low back pain **(C,D)**, and a patient with radiculopathy **(E,F)**. **(A,C,E)**: Sagittal T2-weighted (T2w) images show the absence of morphologic signs of relevant IVD degeneration **(A)**, substantial dehydration at the L4/L5 segment **(C)** and the L5/S1 segment **(C,E)** accompanied by extrusion at the L4/L5 segment **(E) (B,D,F)** Sagittal glycosaminoglycan Chemical Exchange Saturation Transfer (gagCEST) images with overlaid color-coded maps to visualize the GAG contents of the IVD segments. Low GAG content is depicted in blue, and high GAG content is depicted in red. The unit of scale on the right is gagCEST effect in %. The lowest values are found in the patient with non-specific low back pain (nsLBP), while the highest values are seen in the asymptomatic volunteer. Reproduced with permission from Frenken et al., (57).

GagCEST is the only technique that directly quantifies glycosaminoglycans, compared to the indirect measurements summarized above. It does so by measuring the exchange of hydroxyl protons between glycosaminoglycans and water molecules and has been demonstrated to be effective in assessing the articular cartilage of large joints in the setting of osteoarthritis and chondral damage (54, 55) with the capacity for improved reproducibility and sensitivity to regional differences in glycosaminoglycan content with refined protocols (55, 56), a major critique of gagCEST. About the spine, a 2014 investigation of 25 subjects with a mean age of 46 years identified significantly lower gagCEST values in the lumbar IVDs with increasing age (55). Findings included a significant negative correlation between glycosaminoglycan content and age in the nucleus pulposus and the annulus fibrosus (55), which were reproduced in a 2015 study of 70 volunteers performed by Muller-Lutz et al. (55). In another 2021 study comparing subjects with radiculopathy or non-specific LBP to healthy volunteers, IVDs of patients with non-specific LBP showed lower gagCEST values than those of the volunteers and IVDs directly adjacent to IVD extrusions in subjects with radiculopathy demonstrated lower gagCEST values than distant IVDs (57). Such results demonstrate the promise this technique offers in correlating glycosaminoglycan content with various spine pathologies and in better characterizing the water content of the IVD with various states of disease (Figure 3). As a still-developing technique, however, GagCEST suffers from high levels of heterogeneity *in vivo*, long scan times, and a low signal-to-noise ratio requiring ultra-high field (7 T) systems that currently make it unfit for widespread clinical application.

#### 5.1.5 MR spectroscopy (MRS)

Imaging with MRS is used to analyze the functional cellular environment of examined tissues by using metabolite levels as biomarkers of ongoing physiologic processes (18). This technique is especially useful during the pathological states of hypoxia, inflammation, dehydration, neovascularization, and neoinnervation that characterize the progression of DDD, described earlier in this review, in which these biomarkers (namely lactate, lipids, alanine, collagen) are highly active. During such states of disease, levels of lactate, lipids, and alanine increase and those of glycosaminoglycans and decrease. By analyzing the ratios of these biomarkers, MRS has the potential to help stage the progression of DDD before gross anatomical changes are visible on conventional MRI techniques. In a 2008 study of 65 discs in 36 subjects (17 with LBP and 19 healthy volunteers), MRS was successfully used to distinguish painful IVDs from asymptomatic healthy discs with high sensitivity and specificity (92% and 97%, respectively) based on changes in the ratios between proteoglycan content and combined lactate, lipid, and alanine level (58). Results such as these suggest that MRS provides accurate and precise information about IVD biochemistry in the setting of pathology and support its potential for non-invasive/provocative detection of painful discs, as seen in DDD. Despite its strong upside potential, application of MRS is limited by low signal-to-noise ratios, motion artifacts, long scanning times, and a general lack of availability (18, 20). Further large-scale feasibility studies and protocol optimization to mitigate these limitations could help streamline the application of MRS and provide a tool with significant potential to improve the staging and clinical prevention of DDD.

### 5.2 Positron emission tomography (PET)

#### 5.2.1 ^18^F- fluorodeoxyglucose (FDG)

**Figure 4:**
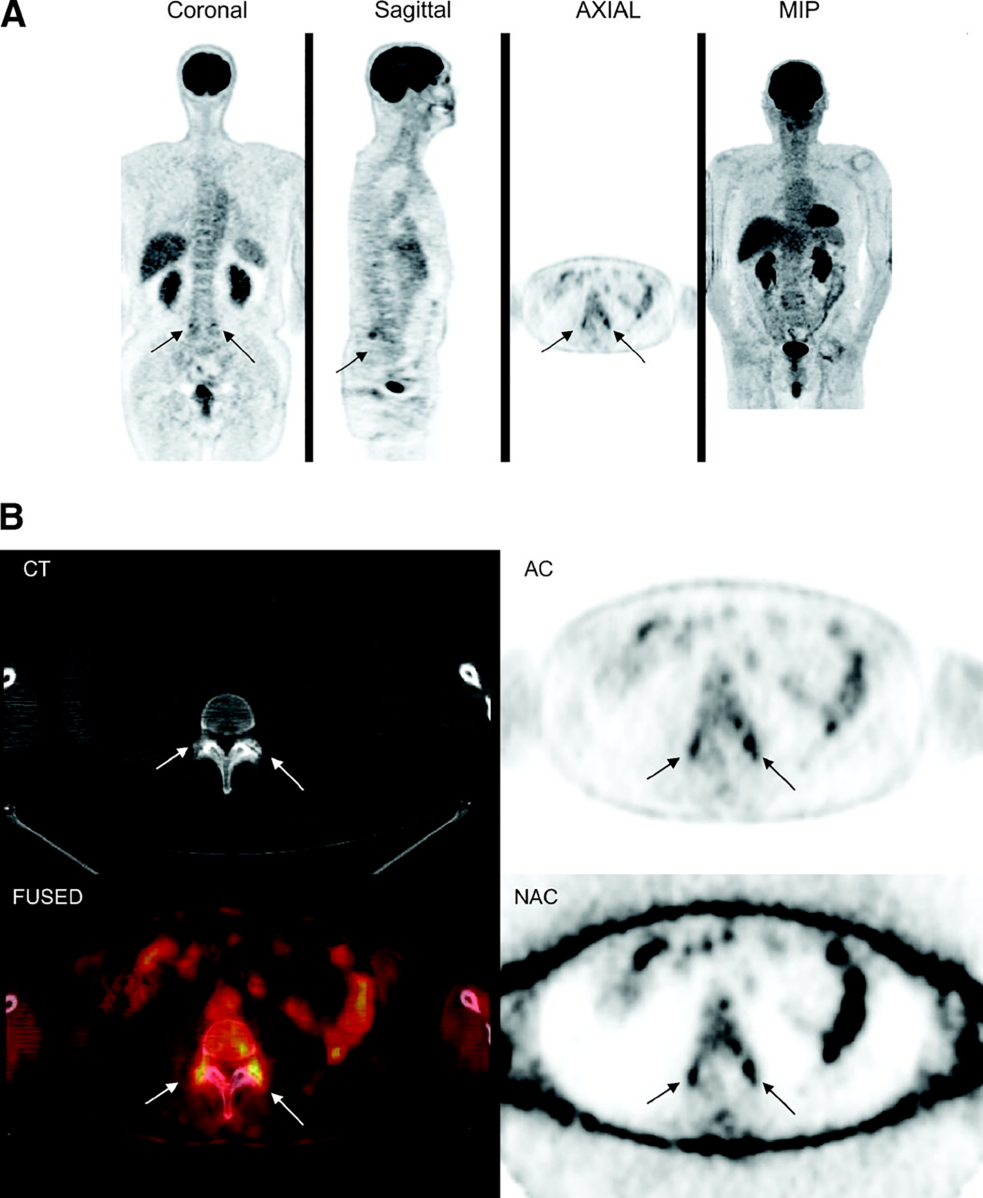
^18^F-FDG PET/CT images of lumbosacral spine show increased 18F-FDG uptake in region of facet joint, corresponding to abnormal findings on CT (arrows). **(A)** Coronal, sagittal, axial, and maximum-intensity-projection (MIP) PET images. **(B)** CT, attenuation-corrected, fused, and nonattenuation-corrected PET images. AC, attenuation-corrected PET image; FUSED, fused CT and PET images; NAC, nonattenuation-corrected PET image. This figure was originally published in *JNM*. Rosen RS, et al. J Nucl Med. 2006;47:1274–1280. © SNMMI. Reproduced with permission. (59).

Research into the utility of FDG-PET/CT in identifying hidden sources of back pain is ongoing and has provided promising data about its ability to evaluate discogenic as well as facetogenic abnormalities of the spine (5, 59–62). In one retrospective blinded review of 150 patients who underwent whole-body FDG-PET/CT for evaluation of increased FDG uptake in the spine and for the presence of degenerative disease, there was significantly more intense tracer uptake in patients with severe degenerative disc changes in comparison to those without obvious degenerative changes on CT (*P* = 0.039) (59). The same was true in those with severe facet joint disease (*P* < 0.0001) (Figure 4) (59). The authors posit that this effect may be explained by the inflammation that accompanies degenerative changed of the spine and suggests that FDG-PET/CT has useful sensitivity of in evaluating hidden pain sources marked by increased metabolic activity, as in inflammation. It is also important to recognize with this outcome that greater tracer uptake does not indicate malignancy and caution should be exercised when interpreting FDG uptake. Notably, the findings also revealed a great deal of variability in the intensity of FDG uptake with regard to the severity of degenerative changes, as some arthritic changes, while severe, may not produce a corresponding severity of inflammatory activity. Regardless of this variability, however, the combined difference between FDG uptake in disc and facet joint disease vs. without was substantial (*P* = 0.0001) and demonstrates well the potential for use of this technique in DDD.

Another study used FDG-PET/CT in 67 patients who presented with back pain and had already undergone XR, CT, and/or MRI that failed to identify a clear cause (60). In patient with back pain and no previous procedures, the FDG-PET/CT showed a sensitivity of 88% in identifying the source of pain as well as positive uptake in all patients with a history of pain after lumbar fusion (60). Another 2017 investigation found similar results in 26 patients by determining that the FDG tracer uptake in patients with LBP was significantly greater at the caudal aspect of the thoracic spinal cord than in patients without pain (*P* < 0.001) (5). Not only are these results consistent with the study above in confirming the ability of FDG-PET/CT to locate discogenic and facetogenic pain sources in the spine, but they reinforce the idea that FDG-PET/CT has a place in localizing more discrete causes of back pain. While PET/CT of the spine is unlikely to supersede MRI as gold standard for imaging in DDD, it is clear it can be a useful adjunct for characterizing less obvious cases of disease, especially in still-progressing cases where inflammation is prevalent. Indications for use will also continue to expand as tracers for new targets involved in inflammation and angiogenesis are developed.

**Figure 5:**
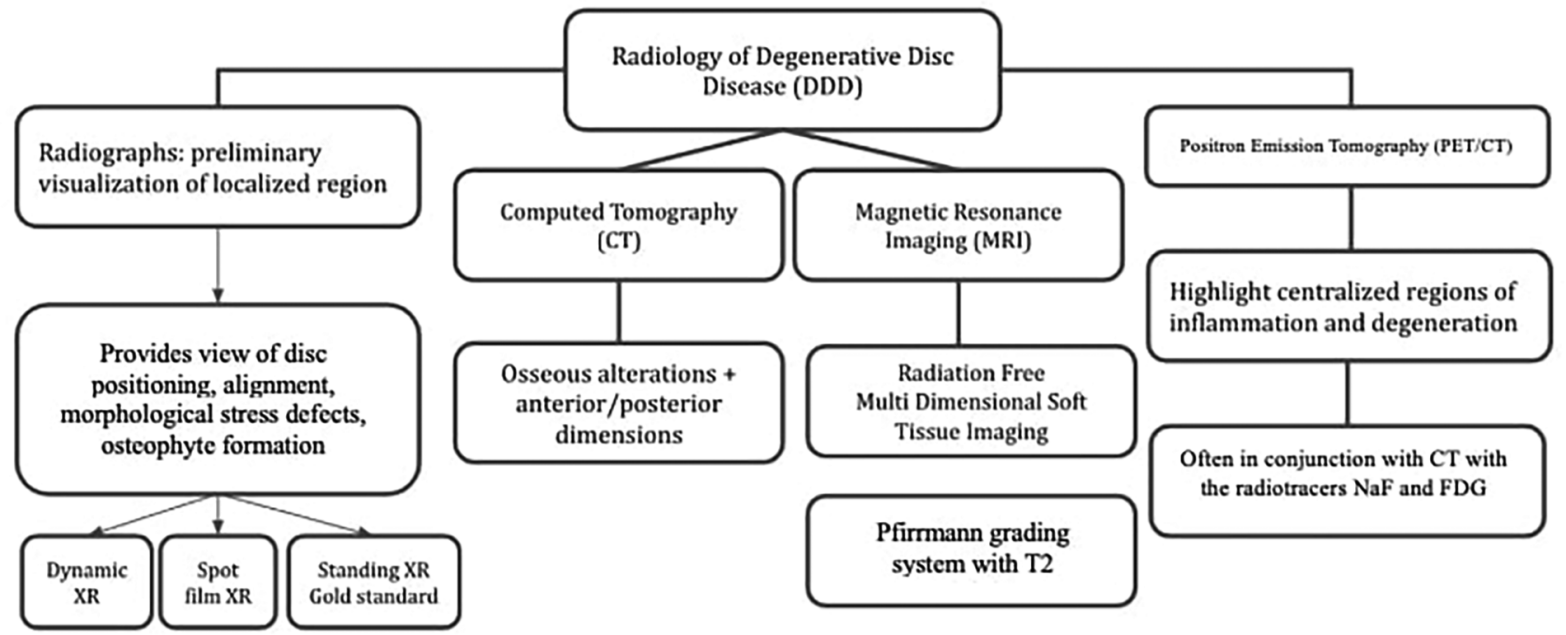
Radiological techniques for degenerative disc disease (1) x-Ray radiographs, (2) computed tomography (CT), (3) magnetic resonance imaging (MRI), (4) positron emission tomography (PET).

FDG-PET along with the other novel advanced imaging techniques, such as PET/MRI, considered in this review, are exciting modalities with strong potential to identify DDD and other sources of LBP early in their clinical courses by locating physiologically active segments of the spine better than is currently possible with conventional imaging techniques. In doing so, they may be able to afford physicians the advanced ability to prevent the progression of LBP with minimally invasive interventions such as intradiscal injections of analgesics, growth actors, anti-inflammatories, intracellular regulatory substances, gene therapies, or synthetic peptides among others still being tested. In any case, such interventions would require early identification of ongoing biochemical processes that precede gross morphological changes, which cannot be seen on XR, CT, or traditional MRI (Figure 5).

#### 5.2.2 ^18^F- sodium fluoride (NaF)

Importantly, 18F-NaF PET has emerged as a potential biomarker for spinal diseases. The osteoblastic activity linked to bone degeneration suggests an association between NaF and age-related spinal degeneration, as documented in the literature. A study indicated that NaF-PET/CT achieved an 84% detection rate in patients experiencing back pain (8), whereas MRI was less conclusive in pinpointing a specific cause in the same patient cohort (21). A retrospective cross-sectional study that examined 18F-NaF PET-CT scans of 88 control volunteers, aged 21–75, revealed that younger participants exhibited significantly reduced 18F-NaF uptake compared to their older counterparts. Furthermore, the extent of degeneration was found to be in correlation with 18F-NaF uptake in both C2-C4 and C5-C7 spinal segments (6).

#### 5.2.3 PET/MRI

Clinically, PET is frequently paired with CT; however, its combination with MRI in hybrid technologies, termed PET/MRI imaging, is also prevalent. PET/MRI offers the unique advantage of combining metabolic and structural assessment, which is particularly valuable when evaluating complex disc pathology such as herniations or fissures. In these cases, extruded disc material can be distinguished from contained herniations, which are wholly held within the annulus and/or posterior longitudinal ligament. These precise categorizations align with recommendations to promote consistent reporting and improved patient outcomes (27). According to a 2018 review in the Journal of Magnetic Resonance Imaging, the hybrid PET/MRI modality boasts enhanced diagnostic sensitivity and specificity (63). It cites a myriad of applications for PET/MRI, encompassing bone and soft tissue sarcomas, multiple myeloma, bone metastases, osteoarthritis, rheumatoid arthritis, osteoporosis, among other degenerative ailments. PET/MRI presents several comparative benefits over PET/CT. These advantages include the elimination of extra ionizing radiation exposure to patients for anatomical localization and the capability to procure additional data, such as diffusion-weighted imaging (DWI). Research involving 9 chronic sciatica patients and 5 healthy controls posited that FDG-PET/MRI can be instrumental in pinpointing pain origins, especially given that the PET and MRI pairing can rectify motion-induced misregistration issues (64). Additionally, in a study examining 42 patients undergoing lower back pain treatment, PET/MRI assessed endplate metabolic activity. The choice of PET/MRI in this context was attributed to its capacity to elucidate both anatomical and functional insights (65). In essence, PET-MRI merges the strengths of both techniques: MRI clarifies PET's anatomical ambiguities, while PET uncovers molecular/inflammatory alterations undetectable by MRI.

### 5.3 Artificial intelligence

Artificial intelligence (AI) and machine learning are rapidly evolving their roles in the realm of imaging and the assessment of spinal diseases. The capabilities of AI extend to enhancing standard spinal imaging procedures, championing preventative strategies, and pinpointing early disease manifestations for prompt interventions. Its potential in diagnosis, prognosis, and outcome prediction for diverse spinal conditions—including scoliosis, spinal fusions, and pediatric LBP—is noteworthy (66–69). A 2020 review delving into AI applications in spinal ailments highlighted the prospects of artificial neural networks in autonomously forecasting Pfirmann grades, Modic alterations, and spinal stenosis grades from MRIs (66). Such advancements could profoundly elevate clinical decision-making. Similarly, Oktay et al. introduced a Computer-Aided Diagnosis (CAD) system for the automatic identification of DDD, factoring in attributes like intensity values, shapes, textures, and context (67). This system operates on a machine learning foundation, encompassing automatic disc labeling and detection, segmentation using active appearance models, and application of Support Vector Machines for training and testing. Through such innovative systems, AI holds the promise of not only amplifying diagnostic precision but also optimizing the treatment and recognition processes of degenerative disc disease. The automation of these detections augments the clinical capacity for interpretation validation.

## 6 The role of MRI

The evaluation of DDD commonly initiates with plain film radiography and is supplemented by standard T1 and T2-weighted MRI scans. These scans help identify structural changes within the nucleus and annulus. Various imaging features, such as the loss of T2-weighted MRI signal, loss of disc bulge or herniation, diminished disc height, vertebral body compromise, and changes in posterior elements, suggest the presence of disc degeneration (17).

The Pfirrmann grading system is the accepted classification scheme for MRI images in the context of DDD. It originally incorporated five grades, determined by factors such as disc structure, the contrast between the nucleus pulposus and annulus fibrosus signal intensities, and disc height on T2-weighted scans (41). Given its widespread application, T2-weighted imaging has become the mainstay of MRI evaluation for disc degeneration. A modified eight-category Pfirrmann grading system has also been proposed to better assess more advanced degenerative disc changes, particularly in elderly populations (70).

MRI has the advantage of being a radiation-free imaging modality, which allows for the comprehensive characterization of soft tissue without compromising multiplanar and multiparametric visualization of spinal tissues (18). However, MRI also has limitations. Specifically, it excels at identifying late-stage disc degenerative changes but is less effective at detecting earlier-stage degenerative features (17). Equivocal scans may result in missed or delayed diagnoses. Moreover, the grading system for MRI images can be both discontinuous and subjective, inadequately representing the complex pathophysiological processes of disc degeneration (18). MRI scans show changes to cellular metabolism that are not in real-time, offering little insight into the functional status of the tissue at the time of the scan (19).

To address these limitations, various studies have examined the utility of alternative MRI techniques, such as Magnetic Resonance Spectroscopy (MRS), T1ρ imaging, and T23Na-MRI. For instance, a 2019 study compared MRS scans of 623 discs in 139 patients with provocative discography (PD) and Pfirrmann grades. The study reported an accuracy of 85%, a specificity of 88%, and a sensitivity of 82% for MRS in herniated discs. For non-herniated discs, these figures were 93%, 93%, and 91%, respectively (58).

T1ρ imaging was found to have a significant negative correlation with disc degeneration (*r* = −0.51, *P* < 0.01) in a study involving ten asymptomatic patients aged between 40 and 60 (46). The technique provides a continuous scale, in contrast to the discontinuous, integer-based scale of T2-weighted imaging that is prone to observer bias (46). Another study involving 105 lumbar discs in 22 subjects revealed that T1ρ and T2 relaxation rates were positively associated with Pfirrmann grades (71). Similarly, T23Na-MRI has been studied for its potential utility. Notably, a 2012 study involving L2-S1 discs in ten asymptomatic subjects found no correlation between sodium imaging and T2 mapping, suggesting that the two could offer complementary insights into DDD (72).

In summary, while traditional MRI provides a valuable, radiation-free technique for diagnosing DDD, it has limitations. The current used grading system is discontinuous and subjective, allowing for observer bias. Moreover, MRI does not offer real-time insights into the status of the disc tissue. Importantly, the technology is better suited for diagnosing late-stage disease than early-stage cases. Current research is thus exploring alternative MRI techniques for a more nuanced and earlier diagnosis of DDD.

## 7 The role of PET

PET is a molecular imaging technique that identifies areas of increased metabolic activity through the use of radioactive tracer molecules, offering potential for early disease detection (73). Commonly used radiotracers include NaF and FDG, which are sensitive to increased calcification and glucose metabolism, respectively (74, 75). These properties make PET potentially useful for diagnosing, treating, and evaluating DDD, as the disease often manifests through metabolic imbalances in its early stages.

Historically, MRI has been the clinical standard for DDD diagnosis. However, PET has shown promise in aiding disease state assessment. Gamie and El-Maghraby conducted NaF PET/CT imaging on 67 patients with back pain (60). Of these patients, 56 (or 84%) showed abnormal NaF uptake in the spine, despite previous routine imaging failing to identify any spinal abnormalities. Moreover, the technique demonstrated high sensitivity in identifying pain sources in subgroups with no prior operative procedures (88%) and those experiencing post-lumbar fusion pain (100%). Rosen et al. conducted a retrospective analysis of FDG-PET/CT scans on 150 adult patients with known or suspected non-brain malignancies (59). The study independently evaluated the increased uptake of FDG in the spine, as well as the presence and severity of degenerative spinal disease (DSD). They found that 58% of the patients exhibited some level of abnormal uptake in the spine, predominantly in the lumbosacral region. However, only 4.7% had findings that were highly suggestive of spinal metastases. These results indicate that FDG-PET is sensitive to non-cancerous abnormal spinal findings of varying severities. Moreover, the study compared FDG uptake levels with the standard CT grading of degenerative disc disease (DDD), finding a statistically significant correlation. Nonetheless, Rosen et al. revealed some limitations in using PET to quantify disease states in patients with degenerative diseases. Despite the statistically significant correlation between FDG uptake levels and CT grading, the strength of the relationship was weak, with a correlation coefficient (*r*) of 0.141. The study also observed a high degree of variability in PET findings, which limits the utility of PET as a quantitative measure of disease state. Furthermore, due to the design of the study, no biopsy proof of inflammation was available, leaving the exact cause of the increased FDG uptake undetermined. This raises questions about the clinical relevance of these findings in the context of DDD.

Lam et al. performed a retrospective analysis aimed at correlating FDG uptake with MRI findings in patients suffering from symptomatic degenerative disease of the lumbar spine, which also included grading for disc degeneration (62). These patients subsequently received epidural steroid injections following their imaging procedures. Consistent with previous studies, patients with symptomatic degenerative disease exhibited increased FDG uptake, primarily in areas corresponding to disc degeneration in the lumbar region (59, 62). A statistically significant correlation of moderate strength was observed between areas of maximal FDG metabolic activity and the locations of the epidural spinal injections. However, the study did not find a significant correlation between the levels of ^18^F-FDG uptake on PET scans and the severity of degenerative findings on MRI. These results underscore further limitations in using FDG-PET for characterizing degenerative disc disease and emphasize the continuing importance of MRI as a guiding tool for treatment decisions.

The limitations of FDG-PET observed in the aforementioned studies may be attributable to the nature of FDG uptake as a measure of metabolic activity (59, 62). Specifically, FDG uptake gauges glucose consumption at sites of degeneration at a particular moment, thereby providing a snapshot of the instantaneous disease state (7, 70). Sites with active inflammation often exhibit significant variability in metabolic states, resulting in fluctuating levels of FDG uptake. In contrast, imaging modalities such as MRI or CT capture the structural consequences of the overall inflammatory process (59). This explains why traditional MRI techniques alone may be inadequate for early detection of DDD, as initial stages of the condition may not yet produce substantial structural changes visible on MRI (17). Specifically, this indicates the potential for PET/MRI to be used in combination given that each modality addresses and lessens the weaknesses of the other, with MRI offering anatomical context and PET offering molecular context.

Consequently, patients with severe findings on MRI or CT scans may exhibit low FDG uptake if their inflammation has subsided (59). Conversely, patients with inconclusive CT or MRI findings might display elevated FDG uptake due to intense, early-stage inflammation. This latter patient population, characterized by negative CT and MRI findings, was the focus of the promising study by Gamie and El-Maghraby (60). This area is likely the most promising avenue for further investigation into the utility of PET for characterizing DDD.

In summary, PET imaging shows potential as a non-invasive method for quantifying the state of DDD, particularly in post-surgical patients (8) via both FDG-PET and ^8^F-NaF PET. However, existing literature has identified significant limitations in this approach. Further research is warranted to explore the use of PET in patients who present with back pain but lack conclusive findings from other imaging modalities. In such cases, PET may complement MRI in enabling earlier diagnosis, characterization, and initiation of image-guided treatment for degenerative disc disease.

## 8 Conclusion

Current research highlights the potential of emerging imaging technologies to enhance the diagnosis and management of DDD. Techniques such as dGEMRIC have been shown to enhance T1 relaxation times, increasing the sensitivity of detecting early disc degeneration (76). T2 relaxation mapping and T1ρ imaging have demonstrated strong correlations with degeneration, providing improved sensitivity over conventional techniques (71). NaF PET/CT and FDG-PET/CT have demonstrated the ability to identify degenerative changes in cervical, thoracic, and lumbar discs, complementing structural findings with metabolic insights (59, 60). Additionally, MRS has shown promise in identifying chemical pain markers, aiding in the management of low back pain (58, 66), while DTI offers valuable data on age-related microstructural disc changes in hydration levels of discs (67–69).

Despite these advancements, significant challenges remain. Validation through further research is essential to address limitations such as the lack of real-time inflammatory markers in PET, challenges in quantitative MRI imaging accuracy, and restricted access to advanced imaging equipment. Addressing these hurdles will pave the way for earlier detection and more effective treatment strategies for DDD.
