# Supplementary material for: The advancement and utility of multimodal imaging in the diagnosis of degenerative disc disease
Source: Front Radiol. 2025 Mar 6;5:1298054. doi: 10.3389/fradi.2025.1298054 (PMC11922948; doi:10.3389/fradi.2025.1298054)
Supplement: Supplementary file 1 [file Datasheet1.pdf]

Supplementary Material

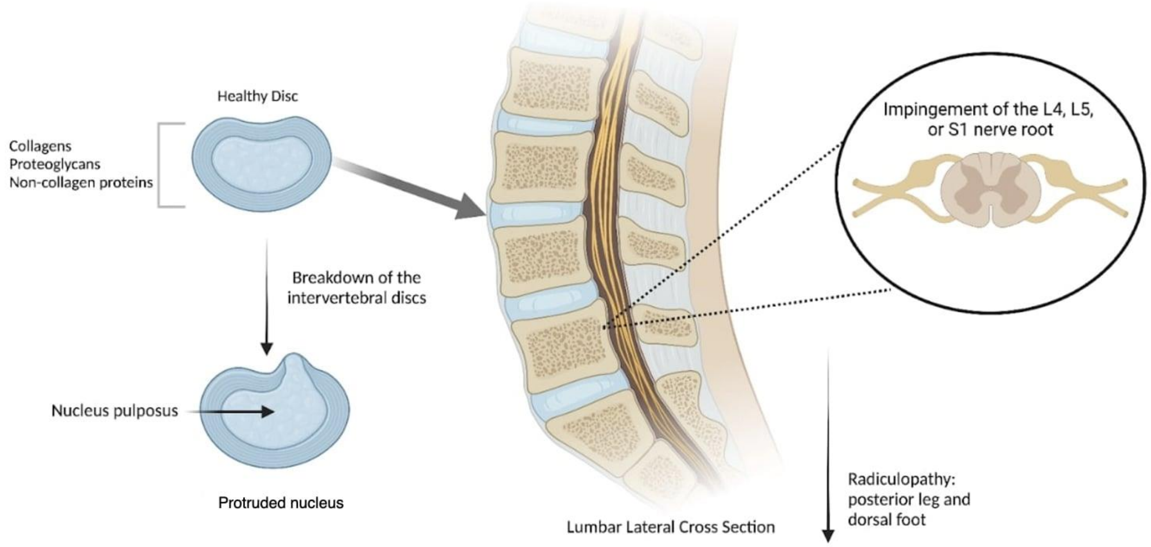

Supplementary Figure 1 Degenerative Disc Disease (DDD) occurs when spinal discs are herniated and the nucleus pulposus is protruded due to mechanical stress, resulting in a compromised structure that induces pain radiating downward. Created with Biorender.

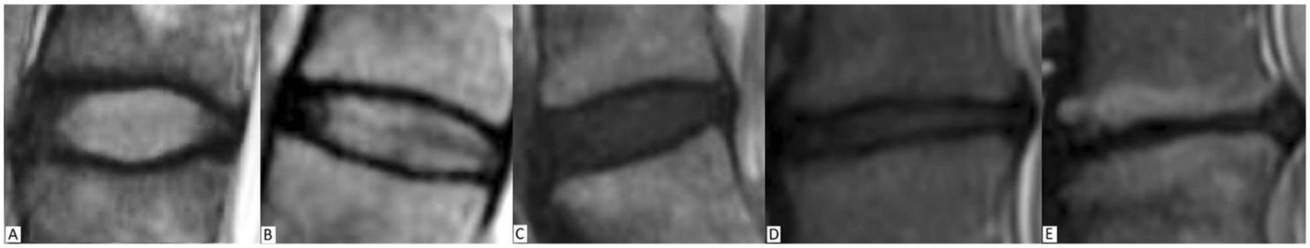

Supplementary Figure 2 Pfirrmann grading system for disc degeneration on sagittal T2 weighted images. a: Grade I, bright and homogeneous disc with clear distinction between nucleus pulposus and annulus fibrosis. Normal disc height. b: Grade II, inhomogeneous disc with horizontal dark band. Nucleus and annulus are clearly differentiated. Preserved disc height. c: Grade III, dark disc with unclear distinction between nucleus and annulus. Disc height is usually normal. d: Grade IV, dark

and heterogeneous disc with decreased disc height. e: Grade V, dark and collapsed disc with no distinction between the nucleus and annulus. Reproduced with permission from Abdalkader et al., 2020. (77)

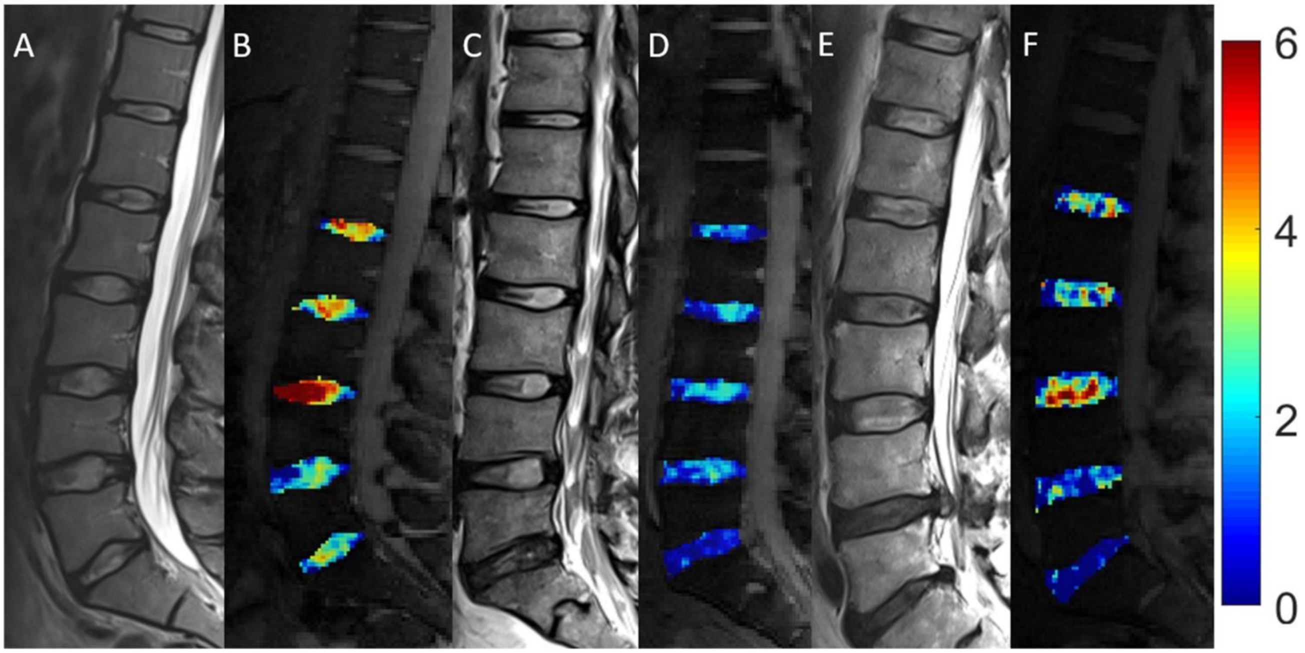

Supplementary Figure 3 Morphologic and compositional imaging findings of lumbar intervertebral discs of an asymptomatic volunteer (A,B), a patient with nonspecific low back pain (C,D), and a patient with radiculopathy (E,F). A, C and E: Sagittal T2-weighted (T2w) images show the absence of morphologic signs of relevant IVD degeneration (A), substantial dehydration at the L4/L5 segment (C) and the L5/S1 segment (C,E) accompanied by extrusion at the L4/L5 segment (E). B, D and F: Sagittal glycosaminoglycan Chemical Exchange Saturation Transfer (gagCEST) images with overlaid color-coded maps to visualize the GAG contents of the IVD segments. Low GAG content is depicted in blue, and high GAG content is depicted in red. The unit of scale on the right is gagCEST effect in %. The lowest values are found in the patient with non-specific low back pain (nsLBP), while the highest values are seen in the asymptomatic volunteer. Reproduced with permission from Frenken et al., 2021. (57)

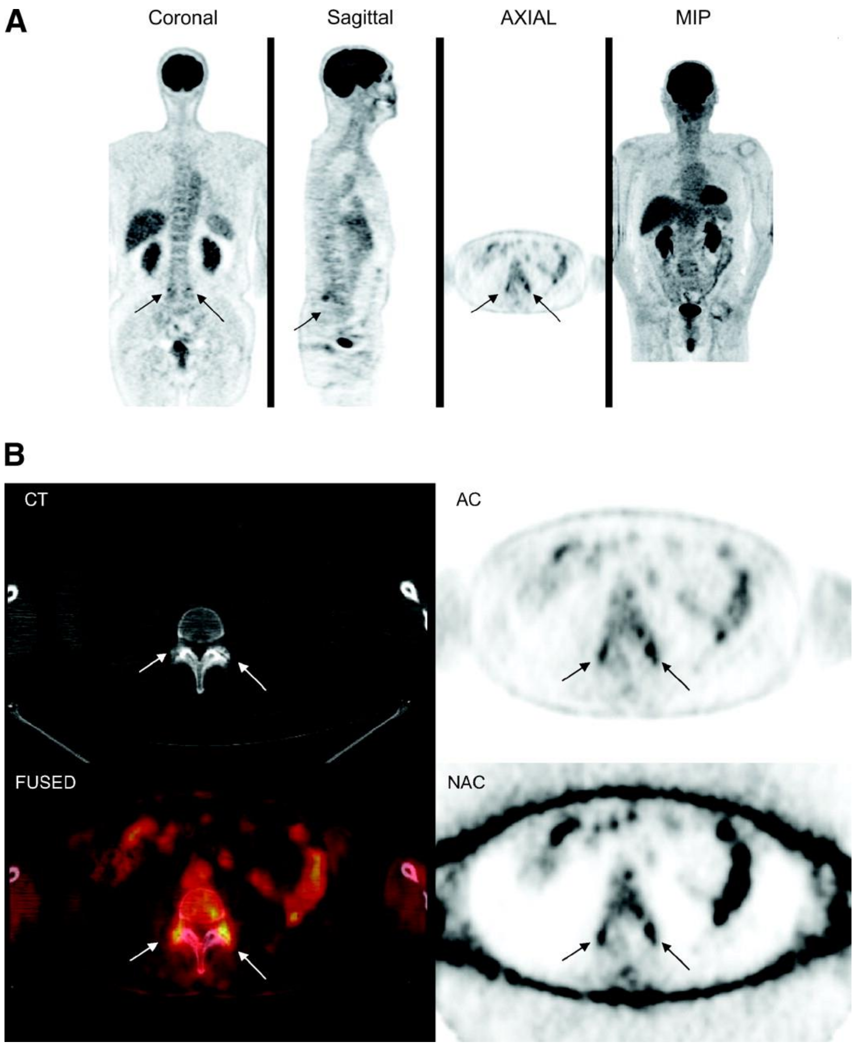

Supplementary Figure 4 ^{18}F-FDG PET/CT images of lumbosacral spine show increased 18F-FDG uptake in region of facet joint, corresponding to abnormal findings on CT (arrows). (A) Coronal, sagittal, axial, and maximum-intensity-projection (MIP) PET images. (B) CT, attenuation-corrected, fused, and nonattenuation-corrected PET images. AC = attenuation-corrected PET image; FUSED = fused CT and PET images; NAC = nonattenuation-corrected PET image. This figure was originally published in *JNM*. Rosen RS, et al. J Nucl Med. 2006;47:1274–1280. © SNMMI. Reproduced with permission. (59)

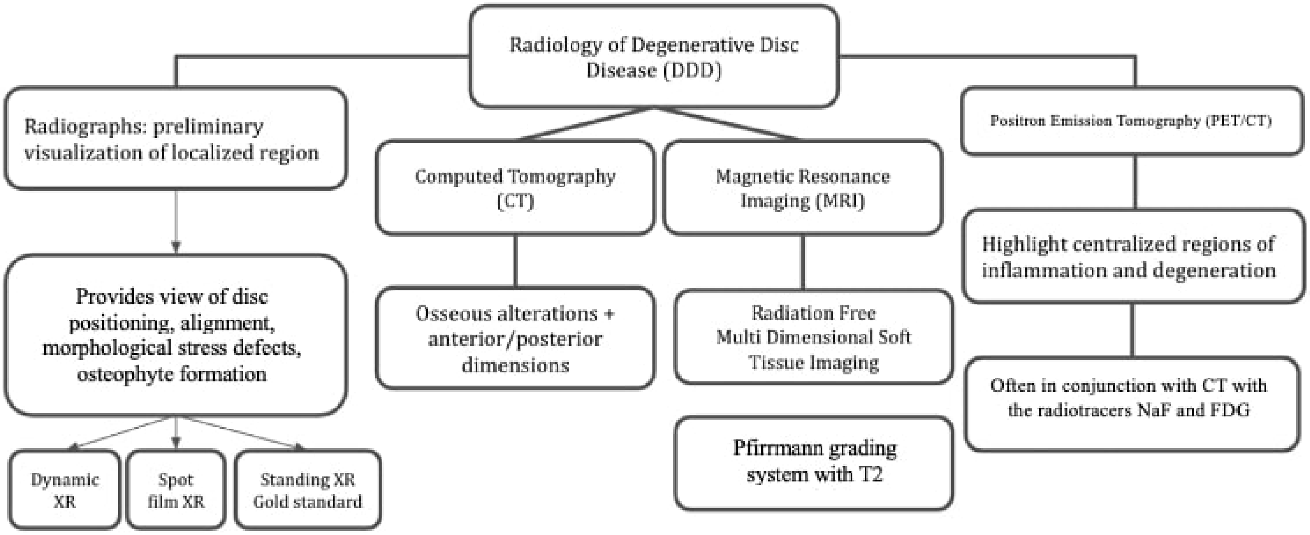

Supplementary Figure 5 Radiological techniques for Degenerative Disc Disease 1) X-Ray Radiographs, 2) Computed Tomography (CT), 3) Magnetic Resonance Imaging (MRI), 4) Positron Emission Tomography (PET)
